# Short- and long-term agreement and reproducibility of 48-hours intraocular pressure measurements in glaucoma patients

**DOI:** 10.1186/s12886-021-02003-4

**Published:** 2021-06-21

**Authors:** Marion Zimmermann, Bert C. Giers, Anna Beck, Katharina Bell, Herwig Zimmermann, Marlene Hechtner, Esther M. Hoffmann, Norbert Pfeiffer, Katrin Lorenz

**Affiliations:** 1 Department of Ophthalmology, Augenklinik und Poliklinik, University Medical Center of the Johannes Gutenberg-University, Langenbeckstraße 1, 55131 Mainz, Germany; 2 University Medical Center of the Johannes Gutenberg-University, Mainz, Germany

**Keywords:** 48-hours intraocular pressure profile, Diurnal and nocturnal intraocular pressure measurements, Pressure fluctuation, Intraocular pressure, Glaucoma

## Abstract

**Background:**

Glaucomatous eyes often show strong intraocular pressure (IOP) fluctuations and individual measurements at different time points are necessary for personalized therapy. To survey IOP variations 48-hours diurnal and nocturnal IOP measurements were performed on two consecutive days. Aims of this study were to investigate the short-term repeatability of 48-hours measurements within one patient’s IOP profile and long-term repeatability between two separate IOP profiles of the same patient.

**Methods:**

A retrospective cohort study was performed evaluating data of 90 glaucoma patients in a German university medical center between 2006 and 2013. All patients underwent two separate diurnal IOP profiles of 48 h. IOP was measured at 8 am, 2 pm, 6 pm, 9 pm using Goldmann applanation tonometry and at 12 midnight using Perkins tonometry in supine position on two consecutive days. Intraclass correlation coefficients (ICC) were calculated to evaluate agreement for the same time points (each time point agreement) and for consecutive measurements within the IOP profiles (between time point agreement). ICC ≤ 0.4 was defined as poor agreement, 0.4–0.75 as moderate and ≥ 0.75 as excellent. Differences between time points were investigated by Bland Altman plots.

**Results:**

Each time point measurements of profile 1 showed moderate to excellent agreement (ICCs 0.62–0.93). There was a moderate to excellent agreement for measurements between time points of profile 1 (ICCs day one 0.57–0.86, day two 0.71–0.90). Profile 2 was performed at a median interval of 12.0 months (quartiles 11.0 to 21.0). Each time point agreements within profile 2 showed ICCs from 0.23 to 0.81. It showed moderate to excellent agreement for changes between time points (ICCs 0.53–0.94). Day two demonstrated ICCs from 0.74 to 0.88. Long term IOP repeatability (over both pressure profiles) showed moderate to good agreement (ICCs 0.39–0.67).

**Conclusions:**

Short and long-term agreement of IOP measurements evaluated by diurnal IOP profiles is moderate to good. Due to mostly moderate agreements, which we believe represent IOP fluctuations, we conclude that it is necessary to perform 48-hours IOP profiles to gain a better overview of the individual IOP fluctuations.

## Background

A major risk factor for the development of glaucoma as well as disease progression is an elevated intraocular pressure (IOP) [1]. IOP is the only therapeutic target we have in the clinical routine. It has been suggested that not only absolute IOP levels but also IOP fluctuations are important for disease progression. Studies show IOP daytime fluctuations in healthy individuals of 1–5 mmHg and an increase of IOP during the night in supine position [2]. In glaucoma patients, daytime fluctuations of IOP can amount to 5–18 mmHg [3]. In order to establish a patient’s individual IOP profile inpatient diurnal pressure profiles over a period of 48-hours (two consecutive days) are performed in our hospital. IOP is measured five times a day either by Goldmann applanation tonometry (GAT) or via Perkins tonometry at midnight. The advanced glaucoma intervention study (AGIS) showed that there is a coherence of IOP fluctuations with peaks of pressure and progression of glaucoma [4, 5]. These peaks may be more easily detected in an inpatient pressure profile than in single office measurements, since it is not limited to office hours and includes measurements during evening and nighttime. Another aspect is the patient’s compliance to medical therapy. During an inpatient pressure profile eye drop application is done under the control of nursing staff.

There are different and contradictory opinions concerning reproducibility of diurnal pressure profiles. In two separate prospective studies including 40 healthy subjects and 47 primary open angle glaucoma (POAG) patients Realini et al. criticized the short and long-term repeatability of diurnal pressure profiles measured by GAT under the aspect of reproducibility between time points of measurements. IOP measurements were performed between 8 am and 8 pm every two hours for one day and at a second day after one week [6, 7]. They showed that the agreement of IOP change between visits was only poor with intraclass correlation coefficients (ICC) ranging from − 0.11 to 0.38 in POAG patients and − 0.16 to 0.49 in healthy subjects.

Barkana et al. described the importance of 24-hours measurements outside normal office hours. In a study with 32 POAG patients, they measured IOP using GAT every two hours from 7 am until 12 midnight. Additionally, Perkins tonometry in a supine position was performed at 6 am. They did not find a sufficient comparability of IOP measured at daytime and in the evening or at night time and concluded that 24-hours monitoring of IOP has a great role for the review of glaucoma progression [8].

Wilensky et al. also demonstrated poor repeatability for IOP measurements. They found that only 34 % of 176 POAG patients as well as 28 % of 55 ocular hypertension (OHT) patients showed a long time reproducibility of pressure values. The pressure measurements were performed with self-tonometry at home five times a day over four to eight consecutive days. Time points of measurements were in the morning after waking up, 12 noon, 4 pm, 7 pm and bevor sleeping, but they had no measurements at night time. Seventy-five of these patients repeated the diurnal self-tonometry after 1 to 45 months [9].

In contrast, Katavisto et al. and De Venecia et al. reported reproducible values for 24-hours pressure measurements in glaucomatous eyes as well as for healthy individuals. In their studies measurements were performed by using Schiotz tonometry [10, 11].

In a prospective study Hatanaka et al. were able to demonstrate short-term reproducibility of IOP measurements in 88 patients with non-treated OHT and POAG. They performed IOP measurements at 8 am, 11 am, 2 pm and 4 pm on two consecutive days. With ICC from 0.80 to 0.86 they concluded that there is an excellent agreement between day one and day two [12].

Concerning the long-term repeatability of IOP measurements Aptel et al. published a prospective cohort study. Analyzing 92 patients with POAG, they performed four diurnal pressure profiles with intervals of six months between every pressure profile. Time points of measurement were 9 am, 10 am, 11 am, 12 am, 2 pm, 3 pm, 4 pm and 5 pm performed with GAT. There was only poor to moderate agreement with ICCs between 0.26 and 0.77 and in conclusion they found no long-term reproducibility of pressure profiles [13].

The aim of this study was to survey the agreement of 48-hours inpatient pressure profiles to detect IOP fluctuations and survey nighttime pressure spikes and the importance of performing nighttime IOP measurements. In order to find the optimal therapeutic procedure and detect IOP fluctuations on time that might cause disease progression we routinely perform these pressure profiles. In this manuscript the evaluation of diurnal pressure profiles concerning short and long-term agreement is described.

To the best of our knowledge, this is the first study that evaluates 48-hours pressure profiles including nighttime measurements in supine position.

## Methods

### Study design

To evaluate short- term and long-term agreement of diurnal IOP profile measurements a retrospective cohort study was performed. Data of 90 glaucoma patients that underwent at least two different diurnal intraocular pressure profiles during two subsequent days in the period of 2006 to 2013 at the Department of Ophthalmology, University Medical Center of the Johannes Gutenberg-University Mainz, were analyzed. Ethics Committee approval was not required for this scientific project, as only routine health data of patients treated in the Department of Ophthalmology, University Medical Center Mainz, have been investigated by retrospective chart review. No so-called third persons have been allowed to have a direct inspection of original health data of individual patients and the analysis and publication has been performed with anonymised data only. A more detailed formal approval is not required, because this type of investigation is regulated by the „Landeskrankenhausgesetz, §§ 36–37“ (State Hospital Law of Rhineland-Palatinate, Germany).

### Patient population and data collection

Patients with the different glaucoma subtypes POAG, OHT, normal tension glaucoma (NTG), pseudoexfoliative (PEX) glaucoma and pigment dispersion glaucoma (PDG) as well as patients with optic discs suspicious for glaucoma were included. Anti-glaucomatous therapy was the same or none in both eyes during one diurnal pressure profile. A change of therapy between the two pressure profiles was possible. Patients that had any IOP lowering surgeries before or during the pressure profile were excluded. The duration of the disease was not taken into account. Furthermore, we did not differentiate whether the patients had received several diurnal IOP measurements in the past or whether the patients were naive to diurnal IOP measurements.

IOP measurements were performed at five time points within 24 h (8 am, 2 pm, 6 pm, 9 pm and 12 midnight) on two consecutive days. IOP was measured with GAT sitting at the slit lamp during the day and Perkins tonometry at 12 midnight when lying in bed. One value was measured for each eye. The wards physician performed daytime measurements at 8 am and 2 pm, at 6 pm the physician on duty measured and another physician measured at 9 pm and 12 midnight. It was not documented which eye was measured first, but routinely the right eye is measured before the left eye, and there was no evaluation if patients received pupil dilation before measurement. IOP measurements were not masked between the different physicians who measured IOP during the IOP profile in this retrospective study. Missing measurements due to absence of patients for this time point of measurement were not imputed.

### Endpoints

Primary endpoints for the evaluation of short-term repeatability were each time point agreement, defined as agreement of measurements between the same time points within the pressure profile (8 am to 8 am, 2 pm to 2 pm, 6 pm to 6 pm, 9 pm to 9 pm, 12 midnight to 12 midnight between day 1 and day 2, respectively) and between time point agreement, defined as agreement of measurements between consecutive time points within the pressure profile (8 am to 2 pm, 2pm to 6 pm, 6 pm to 9 pm, 9 pm to 12 midnight within day 1 and day 2 respectively as well as 12 midnight to 8 am between day 1 and day 2). These measurements were performed for IOP profile 1 and 2.

As secondary endpoint, long-term agreement of profile one and profile two, each time point agreements were performed between the same time points between day 1 at profile 1 and day 1 at profile 2. The same was done for days two between profile one and two.

### Statistical analysis

Patient characteristics were summarized using descriptive statistics. Categorical variables were described using absolute and relative frequencies. Normally distributed continuous variables were described by means and standard deviation otherwise by median and quartiles. To quantify the agreement of measurements, intraclass correlation coefficients (ICC) were calculated stratified for side (left/right eye) using two-way random models. ICC ≤ 0.4 was defined as poor agreement, 0.4–0.75 fair to good (moderate) agreement and ≥ 0.75 as excellent agreement after Landis and Koch scheme [13]. Sample size calculation for the primary endpoints estimated that a minimum of 89 subjects were needed to achieve at least 80 % power to detect an intraclass correlation of at least 0.4 with Bonferroni adjusted significance level of 0.0018. Secondary endpoint analyses were regarded as explorative. In addition, we employed Bland Altman Plots to evaluate the agreements between the IOP measurements and to investigate the existence of any systematic difference between the measurements. Analyses were performed using SPSS 22.0 (SPSS Inc., Chicago, Illinois, USA). Sample size was calculated using R 3.1.0 (R Foundation for Statistical Computing, Vienna, Austria).

### Institutional Review Board

IRB/Ethics Committee ruled that approval was not required for this study.

## Results

### Description of the sample and IOP profiles

Data of 90 patients with glaucoma were included in this study (Table 1). Thirty-seven (41 %) patients included in the study were suffering from POAG, 16 (18 %) patients had a history of OHT, 21 (23 %) patients presented with NTG, 5 (5 %) patients with PEX glaucoma, 4 (4 %) patients with PDG and 7 (8 %) patients belonged to the glaucoma suspect group (evaluated by clinical appearance of the optic nerve head). Due to the inhomogeneous group sizes and the small numbers in some groups, no subtype evaluations were performed. All subjects were Caucasian. 62 % of the patients were female. The mean age was 60.9 ± 11.8 years for females (range 17–84 years) and 60.9 ± 13.2 years for males (range 20–80 years).

**Table 1:** Patient demographic information and medication in intraocular pressure profile 1 and 2

Variable	Profile 1	Profile 2
Mean age, years	60.9 ± 12.4	62.2 ± 10.4
Sex		
Female (%)	62	62
Male (%)	38	38
Number of medications, n (%)		
0	19 (21.1)	16 (17.8)
1	19 (21.1)	22 (24.4)
2	20 (22.2)	16 (17.8)
3	17 (18.9)	20 (22.2)
4	15 (16.9)	16 (17.8)
Medications used, n (%)		
Prostaglandins	51 (56.6)	59 (65.5)
ß-blockers	44 (48.8)	48 (53.3)
Carbonic anhydrase inhibitors	47 (52.2)	46 (51.1)
Adrenergic agonists	25 (27.7)	29 (32.2)

During one diurnal IOP profile patients had equal anti-glaucomatous therapy on both days and there was no change in medication within this profile. Thirty-five patients had a change in therapy from profile 1 to profile 2.

Seventy-five patients (83 %) were phakic at the first diurnal IOP profile of which two received cataract surgery before measurement of the second diurnal IOP measurement (73 phakic patients at profile 2). All other patients were pseudophakic in both eyes.

The average IOP measurements in both pressure profiles ranged between 13.1 mmHg to 19 mmHg. The IOP during profile 1 was highest at 12 midnight. IOP values for both pressure profiles are demonstrated in Table 2. The median time interval between IOP profile 1 and 2 was 12 months (quartiles 11 to 21 months). The earliest follow-up profile took place after two months; the latest after 70 months. In IOP profile 2 the highest values were measured at 8 am on the second day.

**Table 2:** Mean intraocular pressure values in pressure profile 1 and profile 2

	IOP Profile 1				IOP Profile 2			
	Day1		Day 2		Day 1		Day 2	
	Right eye	Left eye	Right eye	Left eye	Right eye	Left eye	Right eye	Left eye
8 am	15.0 ± 4.1	14.9 ± 3.8	15.0 ± 4.0	15.7 ± 4.1	15.7 ± 2.1	19.0 ± 4.2	13.7 ± 3.8	13.7 ± 3.8
2 pm	16.0 ± 3.2	15.7 ± 3.3	15.7 ± 3.6	15.6 ± 3.3	13.9 ± 3.9	14.9 ± 4.2	14.5 ± 3.8	14.6 ± 4.0
6 pm	16.1 ± 3.7	16.3 ± 3.7	15.3 ± 3.4	16.0 ± 4.5	14.6 ± 4.0	14.7 ± 4.4	14.2 ± 3.8	13.8 ± 4.3
9 pm	15.4 ± 3.6	15.1 ± 3.3	14.5 ± 3.4	15.0 ± 5.0	14.1 ± 4.2	14.8 ± 5.8	13.1 ± 3.8	13.5 ± 4.8
12 pm	17.1 ± 4.3	17.1 ± 4.2	15.8 ± 4.2	16.0 ± 4.5	14.3 ± 4.9	14.9 ± 4.7	13.3 ± 3.4	13.8 ± 3.8

### Short-term reproducibility

#### Each time point agreement (within profiles)

**Fig. 1:**
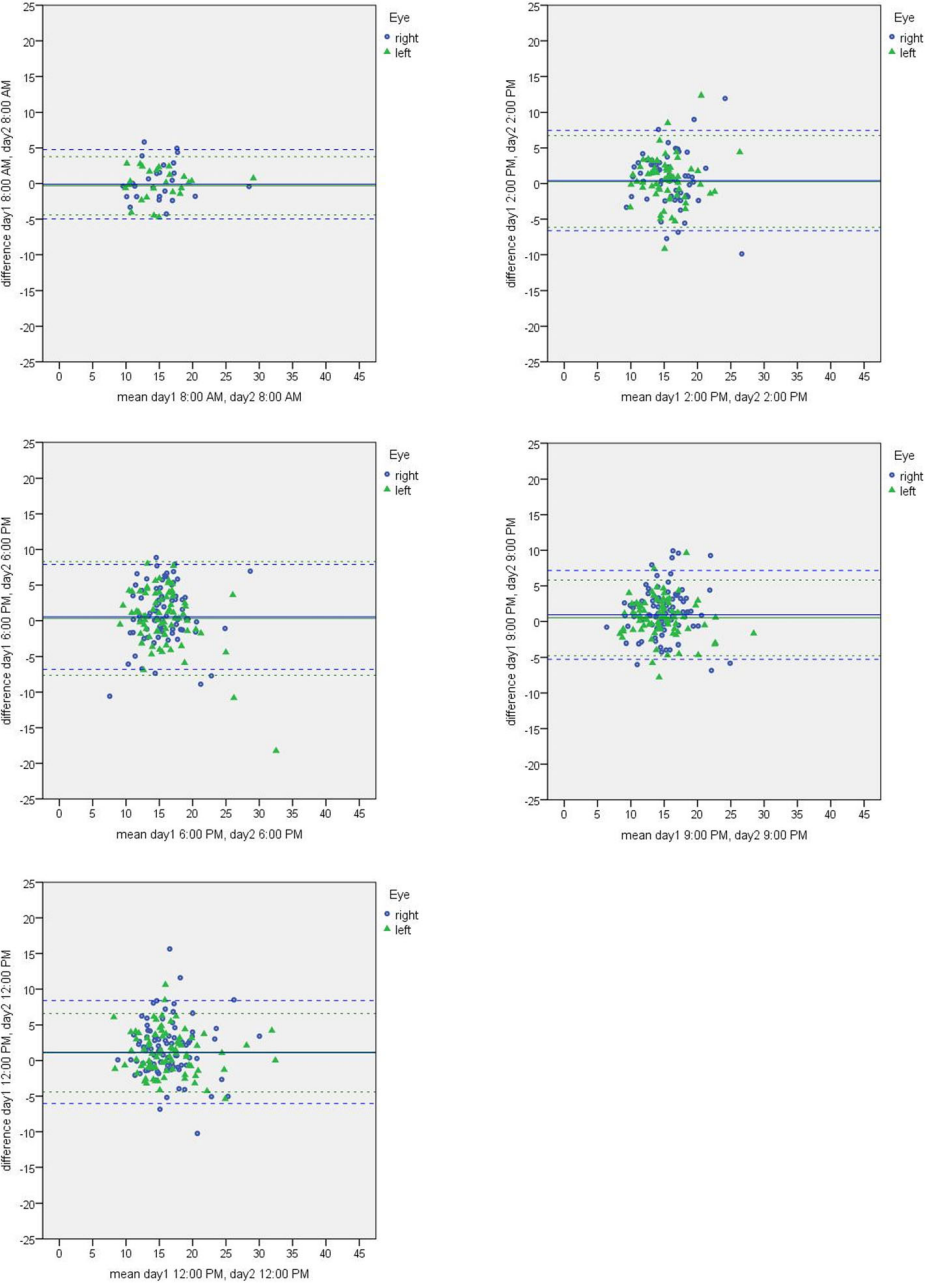
Bland Altman plots for each time point agreement in intraocular pressure profile 1 (8 am to 8 am, 2 pm to 2 pm, 6 pm to 6 pm, 9 pm to 9 pm, 12 midnight to 12 midnight between day 1 and day 2, respectively)

The IOP measurements of each time point between day 1 and day 2 for both pressure profiles are illustrated in Table 3. The ICCs ranged from 0.62 to 0.93 at profile 1 and 0.23 to 0.81 at profile 2. Figure 1 shows exemplary the Bland Altman plots for the comparison of each time point in pressure profile 1. Most values are inside the limit of agreement of the 95 % confidence intervals. The largest distribution appears in the midnight measurements.

**Table 3:** Intraclass correlation coefficients (ICCs) of each time point comparison of intraocular pressure profile 1 and profile 2

	IOP Profile 1^1^		IOP Profile 2^1^	
	Right eye	Left eye	Right eye	Left eye
8 am	0.91	0.93	0.23^2^	0.69^3^
2 pm	0.62	0.68	0.73	0.75
6 pm	0.62	0.67	0.81	0.81
9 pm	0.75	0.81	0.79	0.79
12 pm	0.76	0.87	0.71	0.75

^1^All *p* < 0.001, if not stated otherwise ^2^*p* = 0.43 ^3^*p* = 0.16

### Between time point agreement (within profiles)

The IOP agreement between time points of measurements for each day at the two pressure profiles are displayed in Table 4. The ICCs ranged in profile 1 from 0.57 to 0.86 at day one and from 0.71 to 0.89 on the second day. In profile 2, ICCs varied between 0.53 and 0.94 at day 1 and between 0.73 and 0.88 at day 2. The IOP agreement of measurements before and after midnight (9 pm to 12 midnight and 12 midnight to 8 am) showed smaller ICC values with 0.57 in profile 1 and 0.53 in profile 2.

**Table 4:** Intraclass correlation coefficients (ICCs) of the comparison between time points of intraocular pressure profile 1 and profile 2 in both eyes for both consecutive days of measurement

	IOP Profile 1^1^				IOP Profile 2^1^			
	Day 1		Day 2		Day 1		Day 2	
	Right	Left	Right	Left	Right	Left	Right	Left
8 am-2 pm	0.73^2^	0.61^3^	0.77	0.82	0.76^4^	0.88^5^	0.79	0.81
2 pm–6 pm	0.86	0.84	0.83	0.82	0.83	0.89	0.88	0.83
6 pm–9 pm	0.67	0.69	0.71	0.89	0.69	0.88	0.73	0.74
9 pm-12 am	0.66	0.73	0.80	0.86	0.83	0.94	0.86	0.85
12 pm-8 am	0.57	0.60	–	–	0.70	0.53	–	–

^1^all *p* < 0.001, if not stated otherwise ^2^*p* = 0.02 ^3^*p* = 0.08 ^4^*p* = 0.14 ^5^*p* = 0.03

For all measurements the ICC values showed mostly moderate agreements with values from 0.23 to 0.93 for each time point comparison and 0.53 to 0.94 for change between time points comparison.

**Fig. 2:**
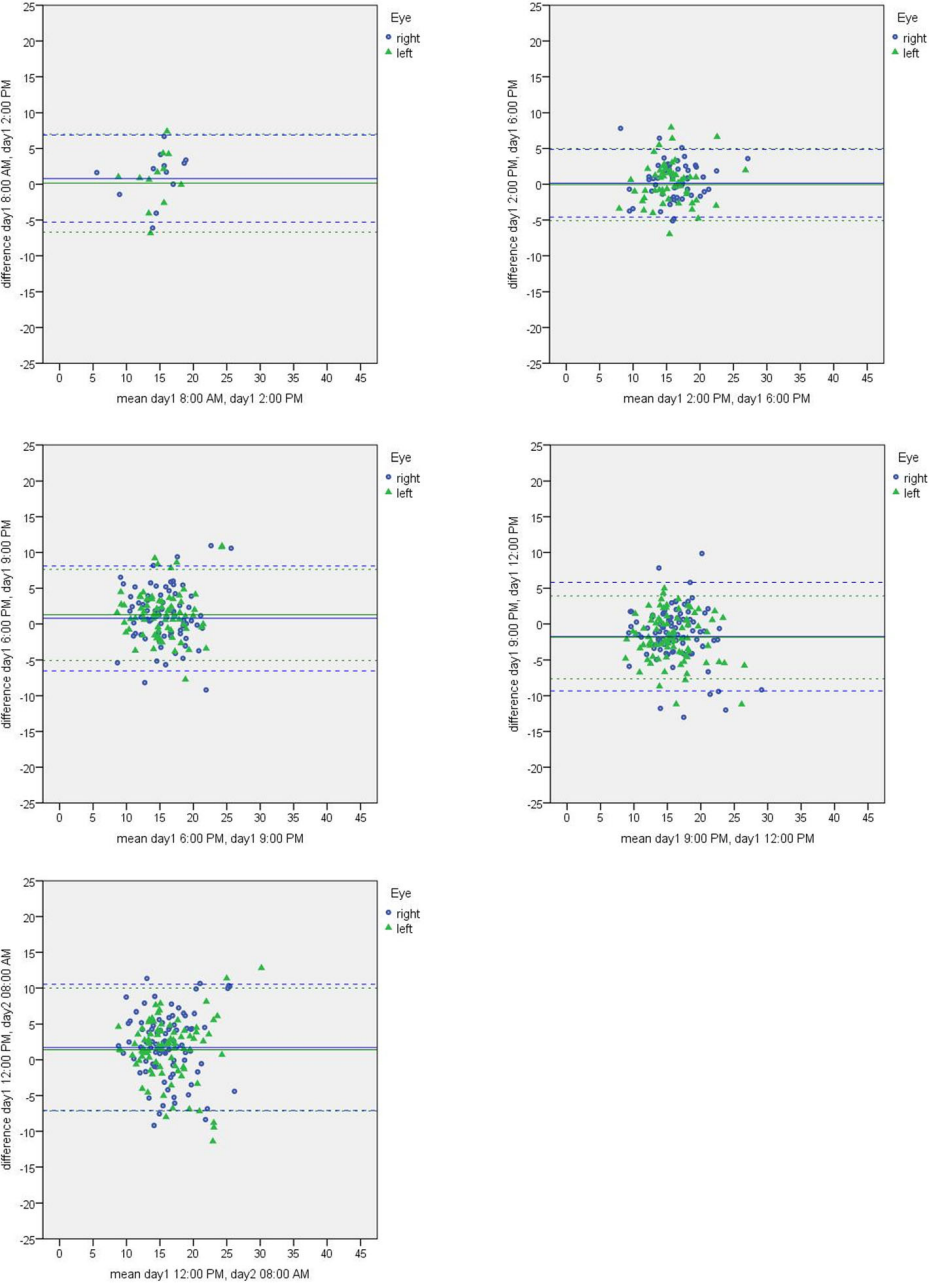
Exemplary Bland Altman plots for the comparison of change between time points in intraocular pressure profile 1 at day 1

Figure 2 shows exemplary the Bland Altman plots for the comparison of change between time points in pressure profile 1 at day 1.

### Long-term reproducibility

#### Each time point agreement (between profiles)

Table 5 shows the long-term comparison of the two pressure profiles in terms of the agreement of IOP measurements at the same time points and days across the two profiles. ICC values in the long-term comparison ranged from 0.49 to 0.86 in the comparison of day one of the two pressure profiles and from 0.39 to 0.69 at day two. Lowest ICC resulted in the comparison of 12 pm at day two.

**Table 5:** Intraclass correlation coefficients (ICCs) in the long-term comparison of each time points between intraocular pressure profile 1 and profile 2 for both eyes and both consecutive days of measurement

	Day 1, profile 1 to profile 2^**1**^		Day 2, profile 1 to profile 2^**1**^	
	Right	Left	Right	Left
8 am	0.86^2^	−^3^	0.62	0.68
2 pm	0.57	0.66	0.57	0.67
6 pm	0.72	0.69	0.69	0.66
9 pm	0.67	0.70	0.56	0.69
12 pm	0.49	0.54	0.39^4^	0.58

^1^all *p* < 0.001, if not stated otherwise ^2^*p* = 0.16 ^3^ICC not available due to insufficient values ^4^*p* = 0.003

Subjects‘ profiles between the high-agreement group and low-agreement group were evaluated regarding glaucoma subtype, lens status, anti-glaucomatous therapy and change of therapy between the two pressure profiles. No significant differences between the groups were found.

## Discussion

 Aim of this study was to evaluate the short-term and long-term agreement and reproducibility between two 48-hours IOP profiles in 90 patients with different types of glaucoma (POAG, OHT, NTG, PEX glaucoma, PDG and glaucoma suspects). Overall, there was moderate to good agreement in the comparison of IOP values within and between the pressure profiles. The discrepancy between the different IOP values demonstrated in the Bland Altman plots showed a large variance. Particularly, when comparing the nighttime measurement at 12 midnight and 8 am the next morning, the variation was larger in comparison to other time points of measurements. These results show that there is not always a predictable pattern of IOP values for every day but the IOP can vary individually. It is important to perform several measurements at different time points and particularly also at nighttime.

There are several studies evaluating IOP fluctuation over time in glaucomatous eyes and healthy individuals, but comparability of the results is often limited. Nevertheless, it is important comparing these studies to find appropriate diagnostic standards for glaucoma patients.

Realini et al. made similar points to the current examination in their study with 40 healthy subjects. Measuring IOP every two hours from 8 am to 8 pm on two visits one week apart they found no repeatable IOP pattern and with these results they concluded that diurnal measurements are important to get knowledge of individual IOP variations and are not replaceable by single-day measurements [6, 7]. It is to attend that they examined healthy subjects in this study. The transfer of their results to our study with glaucoma patients may be limited. In a second study, Realini et al. surveyed 47 patients with POAG using GAT. The study design was copied from the above-mentioned study with healthy subjects, IOP measurements were performed at two separate visits, which were one week apart. As a result, these 47 individuals did not manifest a repeatable diurnal pressure pattern from day to day in the comparison of same time points of measurements in the two visits [6, 7]. In both studies they evaluated the difficulty by building the ICCs (-0.11 to 0.38 for POAG patients, -0.16 to 0.49 in healthy subjects) of IOP values comparable to our study. It was the first study that evaluated the IOP values in diurnal pressure profiles with Goldmann applanation tonometry.

In contrast to these studies, we analyzed IOP measurements performed over two consecutive days. Furthermore, the time points of measurement varied in comparison with previously mentioned studies by examining the patients at 8 am, 2 pm, 6 pm, 9 pm and 12 midnight. Realini et al. performed two additional measurements at daytime but, in contrast, no nighttime measurements were performed. In their studies IOP measurements were performed by a certified ophthalmic technician.

Various studies demonstrate that IOP at night and in a supine position shows higher values than at daytime [14, 15]. That is one reason why it is important to perform the nighttime measurements in glaucoma patients to detect individual IOP fluctuations and to adapt the anti-glaucomatous treatment to this IOP behavior.

In the current study, IOP also showed highest values at 12 midnight in pressure profile 1 at both days of measurements. The ICCs in the comparison between time points around midnight (9 pm to 12 midnight and 12 midnight to 8 am) were only moderate. In the second night, IOP values were lower in comparison to the first night of measurements. An explanation for these values can be given in an increased patient`s compliance with consistent application of anti-glaucomatous medication during hospitalization.

A limiting factor concerning elevated IOP values after waking patients at nighttime while sleeping is partly due to stress-related artifacts by waking up patients for the examination [16]. Additionally, nighttime measurements were performed using Perkins tonometry due to the fact that GAT measurements are not possible in a supine position. Goldmann applanation and Perkins tonometry IOP measurements are known to be comparable. Several studies come to the conclusion that these two applanation methods of IOP measurement are conforming and therefore we are convinced that the validity of our results is given [17, 18].

Another limiting factor in the presented study could be the interobserver variability due to different physicians performing the day and nighttime measurements. Considering the literature this confounder can cause differences in IOP around 2mmHg [19]. In the present study IOP measurements were performed by different experienced examiners. The intraobserver variability can also influence the measured IOP values. Studies were able to demonstrate that measurements performed by the same examiner at different time points can lead to variation of IOP values with an IOP variation as much as 2 mmHg [19, 20].

In the presented case, we included six different subtypes of glaucoma. Realini et al. only included healthy individuals in the first study and patients with POAG in the second study. That is another limitation in comparability of the studies. It is known that glaucoma subtypes show differences in IOP manifestation. For example, OHT patients mostly have higher IOP levels but are infrequently treated with anti-glaucomatous therapy. NTG are characterized by lower IOP levels [21]. PEX glaucoma is known for its high IOP fluctuations [22]. This fact has to be taken into account when evaluating the data. The variety of glaucoma subtypes in our study may reduce the validity of this analysis.

Hatanaka et al. examined 88 patients with OHT and POAG on two consecutive days in a prospective study. They performed IOP measurements at 8 am, 11 am, 2 pm and 4 pm. With ICCs from 0.80 to 0.86 they concluded that there is an excellent agreement between day one and day two [11]. Because of the related study design with measurements on two consecutive days this study seems to show a good comparability to our examination. However, it is to annotate that the patients in the study of Hatanaka did not obtain any anti-glaucomatous therapy and had no surgeries in the past. A topical treatment can generate different IOP profiles and mask suspicious pressure fluctuations [23, 24].

In the current study as well as in the survey of Realini et al. most patients received anti-glaucomatous therapy and there was no differentiation if patients had monotherapy or combined therapy in the data evaluation. In our study, we surveyed patients with different topical therapies. There was every kind of eye drop combination and some patients did not use any anti-glaucomatous therapy. This treatment variety must also be considered in the critical evaluation of the study and may be able to reduce the validity of this evaluation. Moreover, in the study of Hatanaka et al. the nighttime measurement is missing which is also true for the other studies discussed so far [12]. Regarding this aspect, we show that nighttime measurements are an important part of diurnal pressure profiles, showing more fluctuation compared to daytime measurements.

Another limitation of this retrospective study was the potential bias, that IOP measurements were not masked to the different physicians performing Goldmann and Perkins tonometry during the IOP profiles. This should be improved in consecutive studies.

A further limitation of our data is given by the fact that the evaluation of the retrospectively collected data showed that at the first day for the measurement at 8 am there were less documented values of IOP measurement (24/25 patients in profile one and 5/5 patients in profile 2). A reason for these missing values was partly the late arrival of the patients in the hospital and the time consuming diagnostic assessments on the first day (visual fields, OCT imaging, fundus photos) that might have led to missed or delayed IOP measurement. The missing IOP values lead to lower power in the respective evaluation of the 8 am measurements at the first days of IOP profiles and should be noted when reading the results. In following studies, this circumstance should be considered by starting the examination at later time points. Another limitation of this retrospective analysis is that various glaucoma subtypes were included. Thirty-seven (41 %) patients included in the study were suffering from POAG, 16 (18 %) patients had a history of OHT, 21 (23 %) patients presented with NTG, 5 (5 %) patients with PEX glaucoma, 4 (4 %) patients with PDG and 7 (8 %) patients belonged to the glaucoma suspect group (evaluated by clinical appearance of the optic nerve head). Due to the inhomogeneous group sizes and the small numbers in some groups, no subtype evaluations were performed. This should be improved in consecutive studies.

At the first day of measurement during one pressure profile over two days IOP values were averagely higher than in the second day. An explanation for this can be given in the patient’s excitement for the hospitalization. They might have had long stressful arrivals and had to start their days early. Another aspect is the compliance in eye drop application. Without the control of nursing staff the daily routine eye drop application may happen inaccurately. During hospitalization anti-glaucomatous medication is applied by nursing staff at fixed time points and there is no distraction for the patients.

Another interesting analysis would be how many subjects showed high fluctuations and if there were any differences in the clinical and ocular data between the high fluctuation-group and the stable group. This analysis will be part of a second manuscript, which is currently under preparation.

When examining the long-term agreement we compared IOP values of IOP profile 1 with values of profile 2 on the two days of measurements. The second pressure profile was performed an average 19 months after profile 1. The comparison of the same time points from day one in profile one to profile two and day two in both profiles showed only poor to moderate agreements (ICCs from 0.39 to 0.86). These values indicate that the long-term reproducibility has to be questioned and repetitions of diurnal pressure profiles can be useful in clinical routine.

These results confirm results of the study of Aptel et al. In the comparison of IOP values on four diurnal pressure profiles at an interval of six months, the authors could not find long-term reproducibility by building the ICCs [13]. Possible confounders in the evaluation of long-term reproducibility can be the different physicians that perform IOP measurements, change in anti-glaucomatous medication and environmental factors such as personal circumstances that vary between the different pressure profiles.

## Conclusions

Performing inpatient diurnal IOP profiles is an important part of glaucoma diagnostics to detect individual IOP fluctuations. Short and long-term repeatability of IOP measurements during diurnal IOP profiles is overall moderate to good. This is to the best of our knowledge the first study evaluating agreement of IOP over a period of 48-hours including nighttime measurement at 12 midnight. Due to mostly moderate agreements we conclude that it is required to perform 48-hours IOP profiles to get a better overview of the individual IOP fluctuations. The comparisons of IOP values between nighttime measurements around 12 midnight with lowest ICC values for these time points demonstrate that nighttime measurements are useful. To detect more IOP variations additional measurements at nighttime could be performed in the future.

## Data Availability

The datasets used and/or analysed during the current study are available from the corresponding author on reasonable request.

## Abbreviations

am: Ante meridiem
ICC: Intraclass correlation coeficcient
IOP: Intraocular pressure
GAT: Goldmann applanation tonometry
NTG: Normal tension glaucoma
OHT: Ocular hypertension
PDG: Pigment dispersion glaucoma
PEX: Pseudoexfoliation
pm: Post meridiem
POAG: Primary open angle glaucoma
